# Histological assessment, anti-quorum sensing, and anti-biofilm activities of *Dioon spinulosum* extract: in vitro and in vivo approach

**DOI:** 10.1038/s41598-021-03953-x

**Published:** 2022-01-07

**Authors:** Engy Elekhnawy, Walaa A. Negm, Mona El-Aasr, Amal Abo Kamer, Mohammed Alqarni, Gaber El-Saber Batiha, Ahmad J. Obaidullah, Heba M. Fawzy

**Affiliations:** 1 Pharmaceutical Microbiology Department, Faculty of Pharmacy, Tanta University, Tanta, 31111 Egypt; 2 Pharmacognosy Department, Faculty of Pharmacy, Tanta University, Tanta, 31111 Egypt; 3 Department of Pharmaceutical Chemistry, College of Pharmacy, Taif University, P.O. Box 11099, Taif, 21944 Saudi Arabia; 4 Department of Pharmacology and Therapeutics, Faculty of Veterinary Medicine, Damanhour University, Damanhour, 22511 Egypt; 5 Drug Exploration and Development Chair (DEDC), Department of Pharmaceutical Chemistry, College of Pharmacy, King Saud University, Riyadh, 11451 Saudi Arabia; 6 Department of Pharmaceutical Chemistry, College of Pharmacy, King Saud University, P.O. Box 2457, Riyadh, 11451 Saudi Arabia; 7 Histology and Cell Biology Department, Faculty of Medicine, Ain Shams University, Cairo, Egypt

**Keywords:** Antimicrobials, Applied microbiology, Bacteria, Biofilms

## Abstract

*Pseudomonas aeruginosa* is an opportunistic bacterium causing several health problems and having many virulence factors like biofilm formation on different surfaces. There is a significant need to develop new antimicrobials due to the spreading resistance to the commonly used antibiotics, partly attributed to biofilm formation. Consequently, this study aimed to investigate the anti-biofilm and anti-quorum sensing activities of *Dioon spinulosum,* Dyer Ex Eichler extract (DSE), against *Pseudomonas aeruginosa* clinical isolates. DSE exhibited a reduction in the biofilm formation by *P. aeruginosa* isolates both in vitro and in vivo rat models. It also resulted in a decrease in cell surface hydrophobicity and exopolysaccharide quantity of *P. aeruginosa* isolates. Both bright field and scanning electron microscopes provided evidence for the inhibiting ability of DSE on biofilm formation. Moreover, it reduced violacein production by *Chromobacterium violaceum* (ATCC 12,472). It decreased the relative expression of 4 quorum sensing genes (*las*I, *las*R, *rhl*I, *rhl*R) and the biofilm gene (*ndv*B) using qRT-PCR. Furthermore, DSE presented a cytotoxic activity with IC_50_ of 4.36 ± 0.52 µg/ml against human skin fibroblast cell lines. For the first time, this study reports that DSE is a promising resource of anti-biofilm and anti-quorum sensing agents.

## Introduction

*Pseudomonas aeruginosa* is an emerging opportunistic pathogen causing many nosocomial infections like wounds, respiratory and urinary tract infections^1^. The prevalence of *P. aeruginosa* in humans is primarily linked to its ability to form biofilms when attached to biotic and abiotic surfaces. Biofilm is a complex population of bacteria present in an extracellular matrix made of lipids, exopolysaccharides (EPS), nucleic acids, and proteins^2^. This matrix can hamper the spread of different antibiotics through the formed biofilm; thus, antibiotic resistance is highly spreading among the bacterial population embedded in biofilms^3^.

Most bacteria, especially *P. aeruginosa*, communicate through quorum sensing (QS), a signalling mechanism between cells^4^. This mechanism requires the development of molecular signals called autoinducers (AIs) which spread in the surrounding environment and interact with their corresponding regulators leading to increasing biofilm formation and development of antibiotic resistance^5^.

The rapid dissemination of antibiotic resistance worldwide has directed the scientific community toward alternative antibiotics like non-antibiotic quorum sensing and biofilm inhibitors. This approach focuses on hindering the communication between bacterial cells without exerting selective pressure on them, therefore reducing the chance for the appearance of multidrug-resistant strains^5^.

Cycad plants are rich in a wide variety of active constituents. *Dioon spinulosum,* Dyer Ex Eichler, is one of the giant Cycads all over the world. Recently, *D. spinulosum* exhibited potent cytotoxic activity against different cell lines. *D. spinulosum* also displayed a promising *in vivo* hepatoprotective activity. Moreover, it presented antimicrobial, antioxidant, and high protective activities against DNA damage^6,7^.

Not much-reported work has been done in Egypt on different plants that show anti-quorum sensing and anti-biofilm activities. However, many Egyptian plants have been investigated for their anti-cancer, anti-diabetic, antioxidant activities. Thus, we decided in this study to examine *D. spinulosum* plant growing in Egypt for its anti-quorum sensing and anti-biofilm activities against *P. aeruginosa* clinical isolates.

## Results

### Minimal inhibitory concentration (MIC)

The antibacterial activity of DSE against *P. aeruginosa* isolates was determined using broth microdilution assay. The MIC values of DSE ranged from 250 to 1000 µg/ml, as shown in Table 1.

**Table 1:** MIC values of DSE against *P. aeruginosa* isolates.

Isolate number	MIC value (µg/ml)	Isolate number	MIC value (µg/ml)
P1	250	P19	250
P2	500	P20	250
P3	500	P21	500
P4	500	P22	500
P5	250	P23	1000
P6	1000	P24	1000
P7	250	P25	500
P8	250	P26	250
P9	1000	P27	500
P10	1000	P28	250
P11	500	P29	250
P12	250	P30	250
P13	250	P31	500
P14	500	P32	1000
P15	500	P33	500
P16	500	P34	1000
P17	250	P35	1000
P18	1000		

### Determination of cell surface hydrophobicity (CSH)

**Figure 1:**
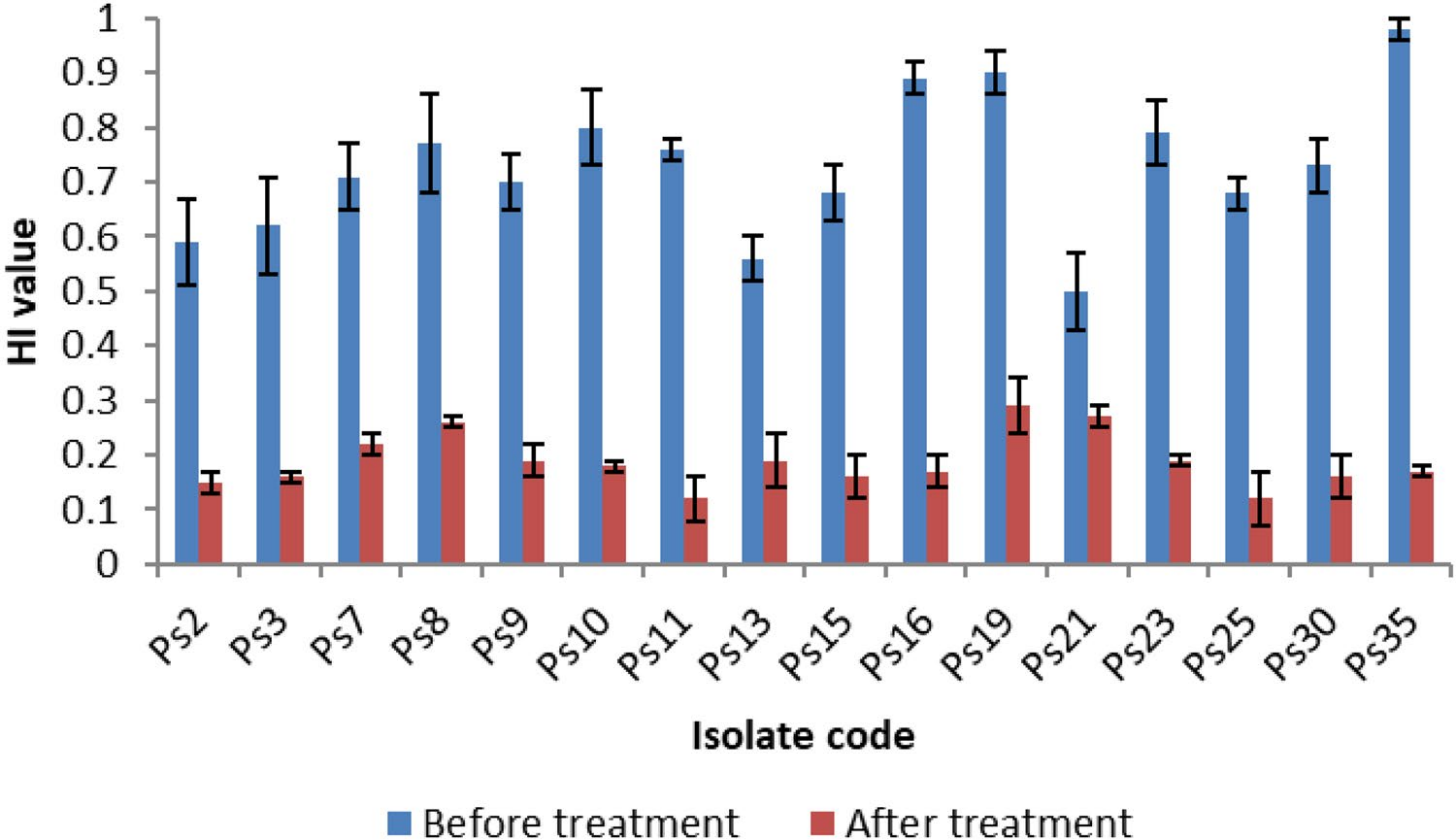
A diagram presenting the significant reduction (p < 0.05) in the hydrophobicity index of 16 *P. aeruginosa* isolates after treatment with DSE.

The CSH was investigated in *P. aeruginosa* isolates by calculating the hydrophobicity index (HI). A significant reduction (p < 0.05) in the values of HI was detected in 45.7% of the tested isolates after treatment with DSE (using concentrations ranging from 125 to 500 µg/ml, which equal to 0.5 MIC values) as displayed in Fig. 1.

### EPS quantification

**Figure 2:**
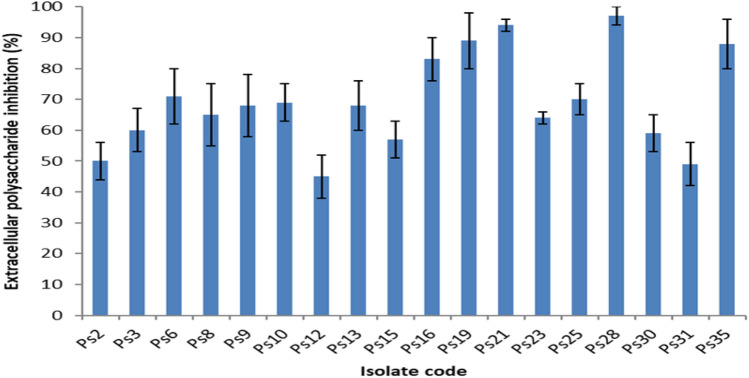
A chart showing the percentages of inhibition of the extracellular polysaccharide production in 18 *P. aeruginosa* isolates after treatment with DSE.

EPS are the main constituents of biofilms; thus, they were quantified before and after treatment with DSE (using concentrations ranging from 125 to 500 µg/ml) by the phenol-sulfuric acid method. A significant reduction (p < 0.05) in the EPS quantity was noticed in 51.4% of *P. aeruginosa* isolates, as shown in Fig. 2.

### In vitro biofilm inhibition assay and counting of colony-forming units (CFU)

**Figure 3:**
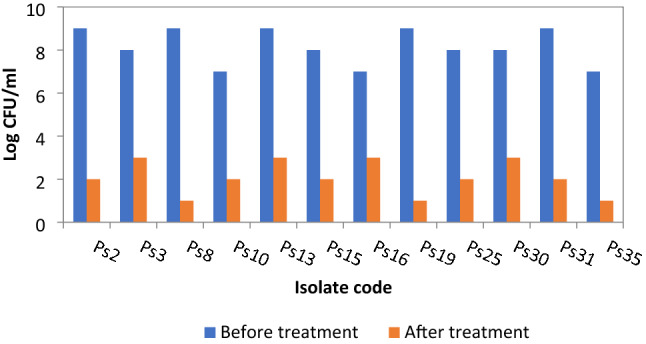
A chart showing significant reduction in the count of CFU/ml of *P. aeruginosa* isolates after treatment with DSE using agar plating technique.

The effect of DSE on biofilm formation was estimated using the crystal violet assay to study whether DSE effectively inhibited biofilm formation by *P. aeruginosa* clinical isolates. We found that DSE decreased the percentage of *P. aeruginosa* isolates, which were strongly and moderately forming biofilm, from 77.1 to 34.3% (as shown in Table 2). The CFU/ml values were expressed as mean ± standard deviation (SD), as presented in Fig. 3. A significant decrease in the number of CFU/ml was detected in 34.3 % of the isolates after treatment with DSE.

**Table 2:** Formation of biofilm by *P. aeruginosa* isolates before and after treatment with DSE (at concentrations ranged from 125 to 500 µg/mL).

Biofilm formation ability	No. of isolates before treatment	No. of isolates after treatment
NBP	6	10
WBP	2	13
MBP	16	7
SBP	11	5

*NBP* non-biofilm producing isolate, *WBP* weak biofilm-producing isolate, *MBP* moderate biofilm-producing isolate, *SBP* strong biofilm producing isolate.

### MTT tetrazolium test

The MTT tetrazolium test was applied to check out the cellular viability of *P. aeruginosa* isolates in the formed biofilm before and after treatment with DSE. A non-significant change (p > 0.05) in the cell viability was observed after treatment with DSE.

### Biofilm examination using compound brightfield and scanning electron microscopes (SEM)

**Figure 4:**
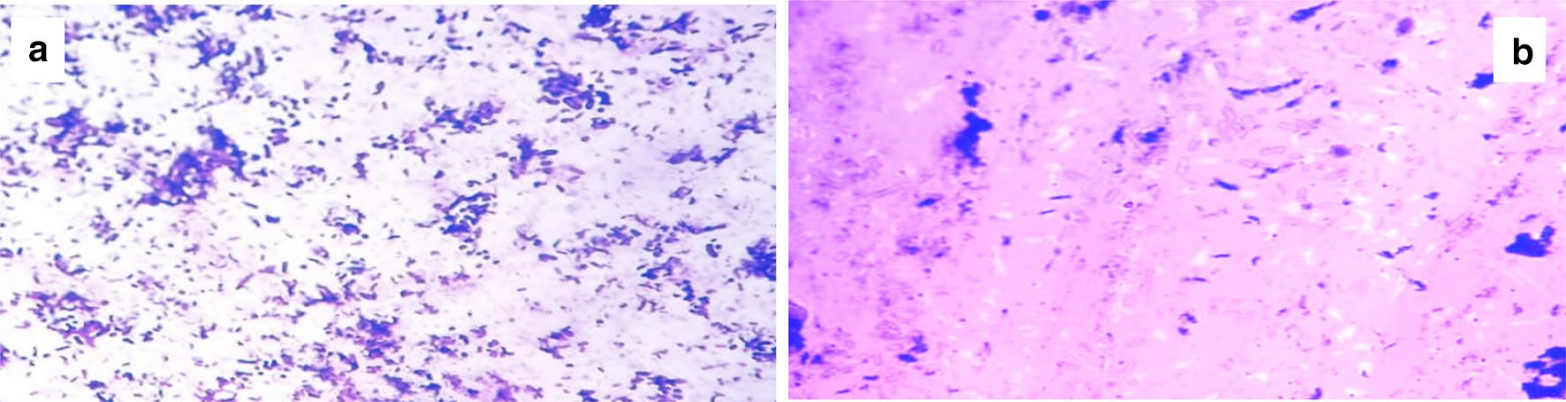
A representative example for the significant reduction in biofilm formation by *P. aeruginosa* isolates using bright field microscope: (**a**) before treatment, and (**b**) after treatment with DSE.

**Figure 5:**
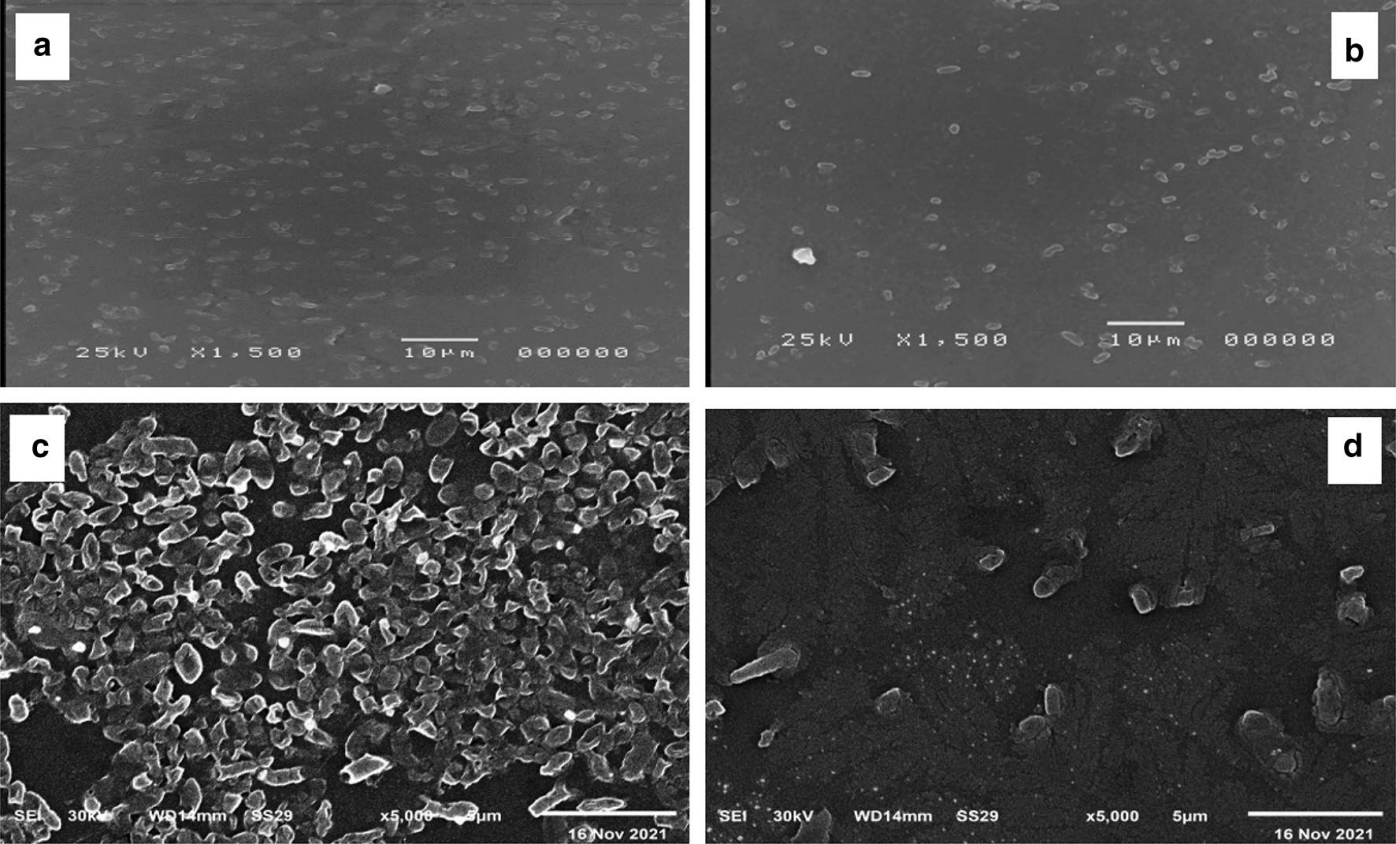
A representative example for the significant reduction in biofilm formation by *P. aeruginosa* isolates using SEM: (**a**,**c**) before treatment, (**b**,**d**) after treatment with DSE.

In order to study the effect of DSE on the biofilm morphology, the formed biofilm by *P. aeruginosa* with (treated isolates) and without (untreated isolates) DSE was examined using a BFM and SEM. A remarkable reduction in the biofilms formed by *P. aeruginosa* was revealed in 15 isolates. A representative example of the visible inhibition of the formed biofilm is shown in Figs. 4 and 5.

### Quantification of inhibition of violacein production

**Figure 6:**
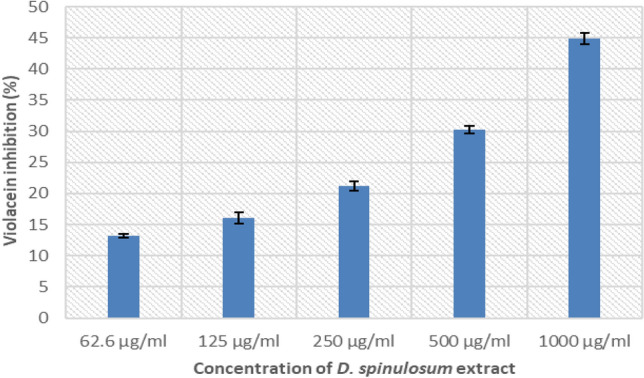
A chart showing a concentration-dependent inhibitory effect of DSE on violacein pigment production by *C. violaceum* (ATCC 12,472).

DSE exhibited an inhibitory effect on the pigment produced by the standard isolate *Chromobacterium violaceum* (ATCC 12472) in a concentration-dependent mode, as shown in Fig. 6. Maximum violacein inhibition of 44.9 ± 0.9% was observed at a DSE concentration of 1000 µg/ml.

### Quantitative RT-PCR

**Figure 7:**
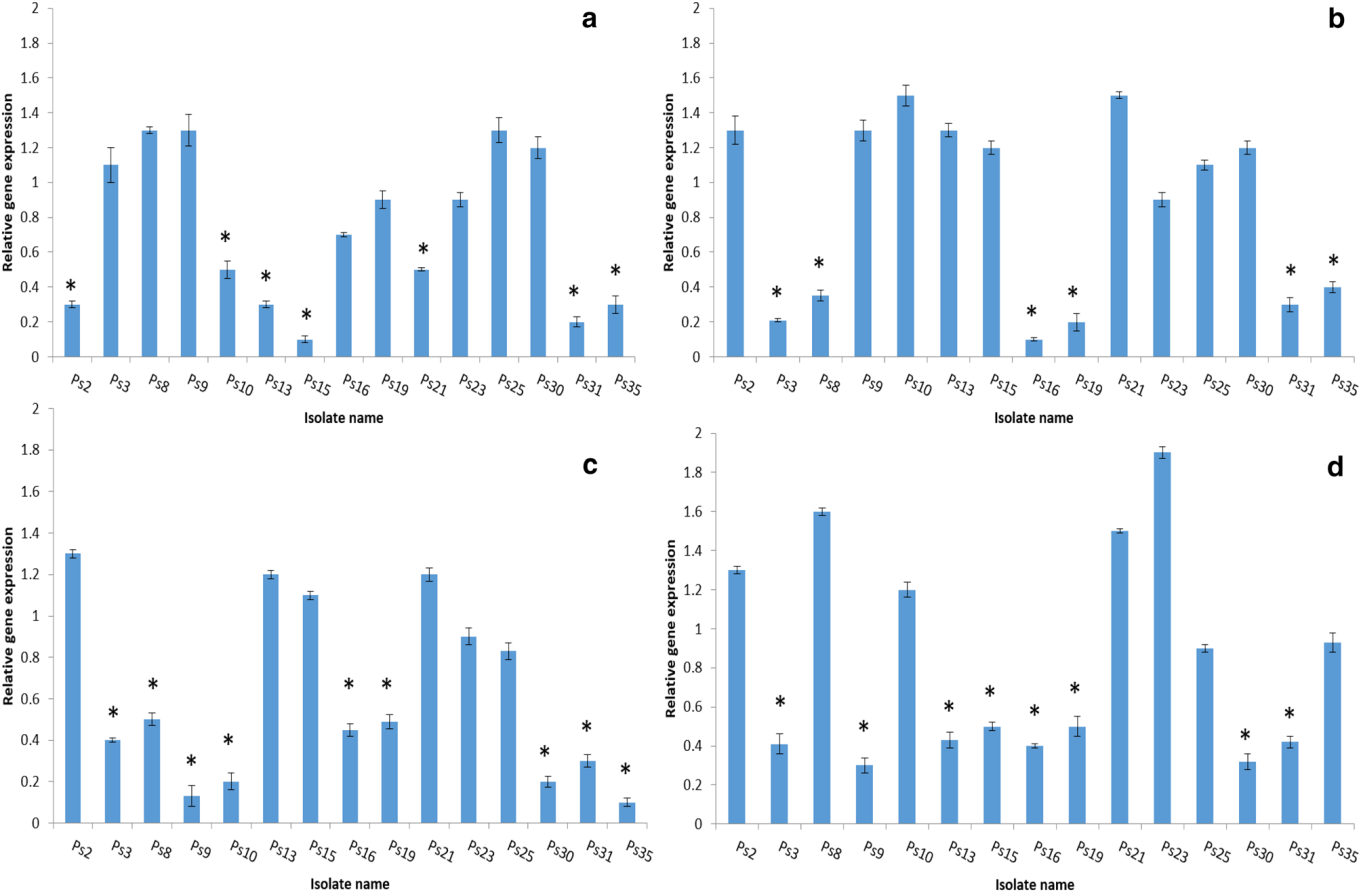
The relative expression of the quorum sensing genes: (**a**) *las*I, (**b**) *las*R, (**c**) *rhl*I, (**d**) *rhl*R of the tested *P. aeruginosa* isolates after treatment with DSE, * indicates the changes in gene expression more than twofold.

**Figure 8:**
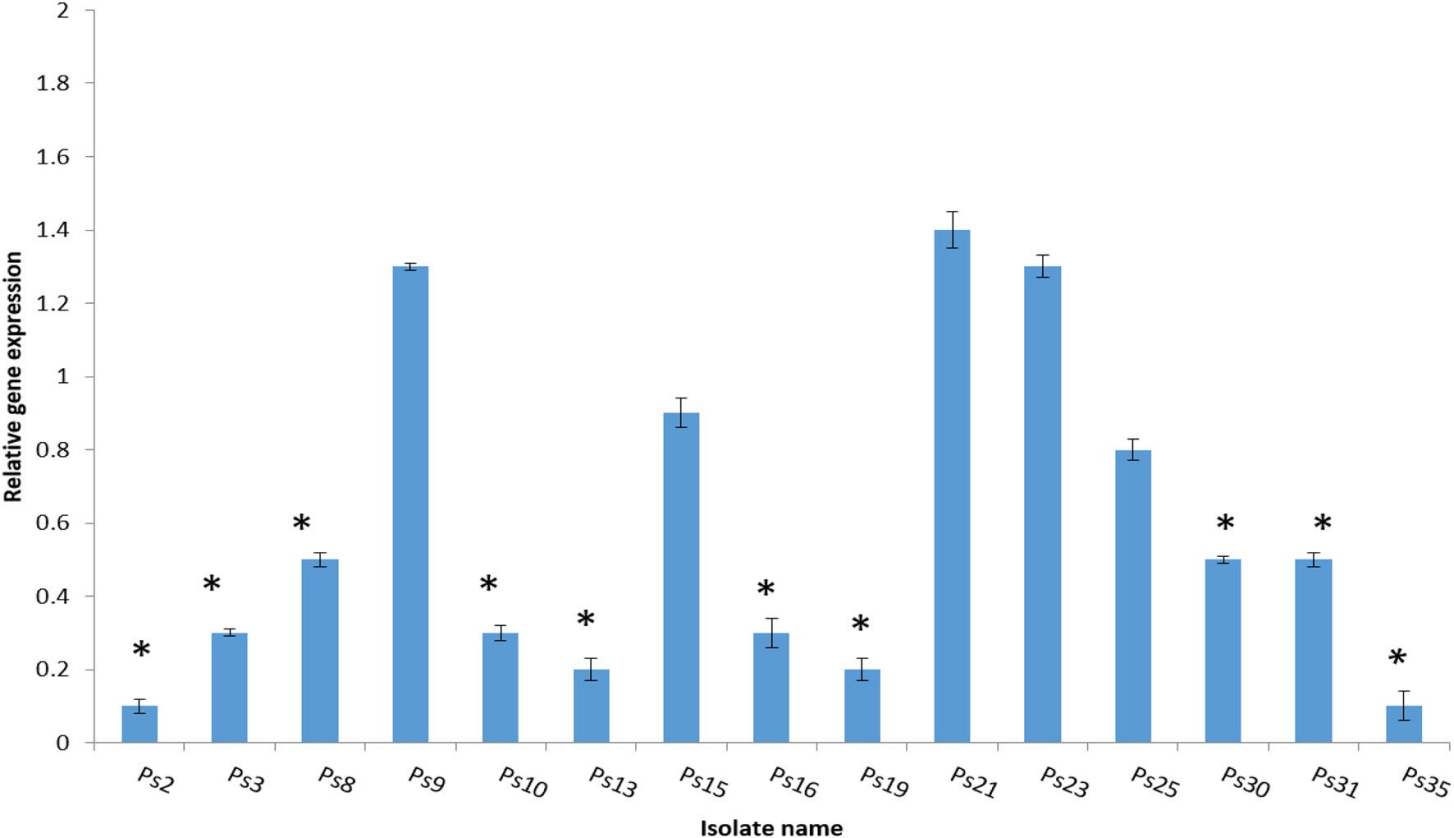
The relative expression of the biofilm gene of the tested *P. aeruginosa* isolates after treatment with DSE, *indicates the changes in gene expression more than twofold.

The relative expression of *las*I, *las*R, *rhl*I, *rhl*R, and *ndv*B genes was studied using qRT-PCR in 15 *P. aeruginosa* isolates (which exhibited a reduction in biofilm formation by the crystal violet test) for better comprehension of the effect of DSE on both QS and biofilm formation as shown in Figs. 7 and 8.

### In vivo biofilm inhibition assay:

**Figure 9:**
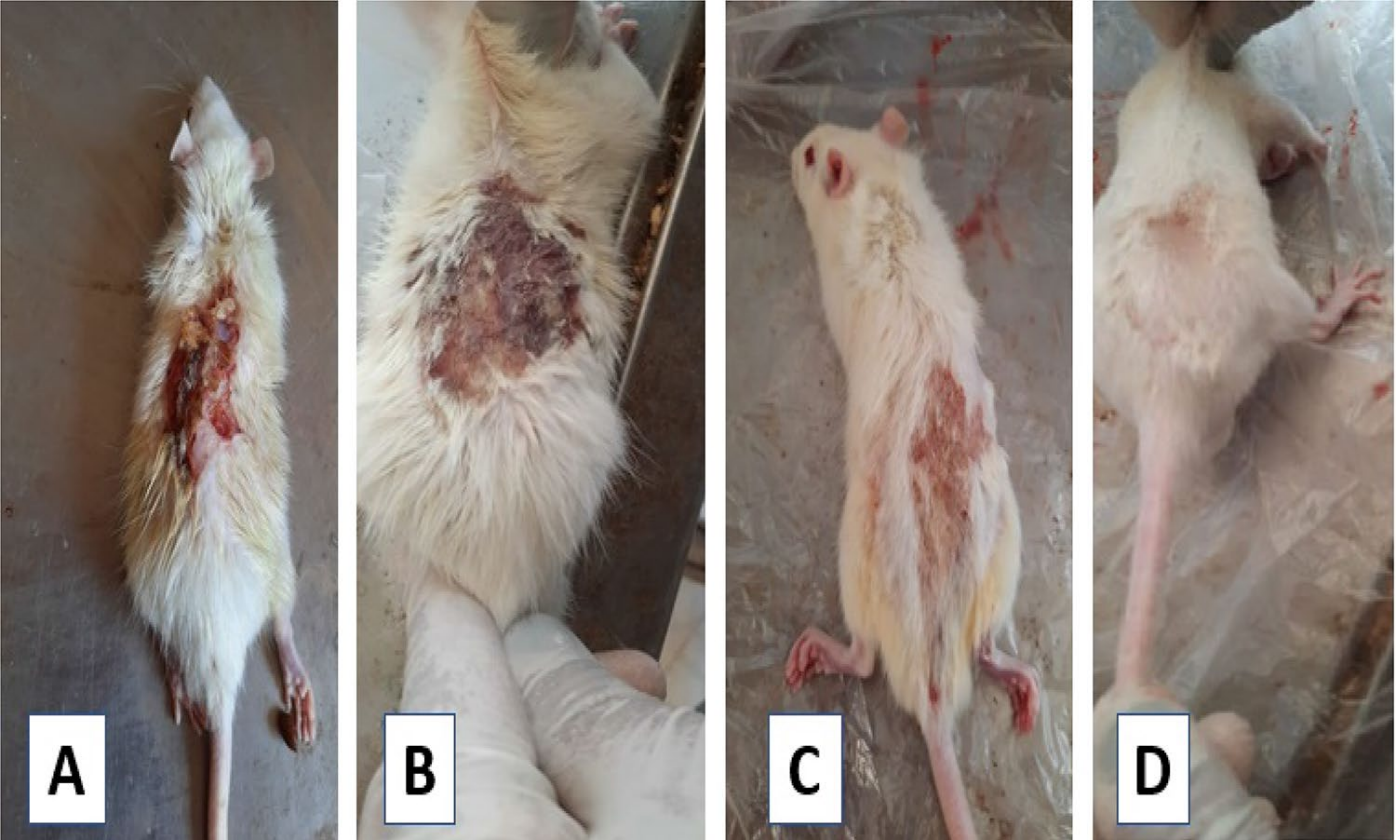
Photographs showing the burned skin of: (**A**) subgroup IIA: the burned area was clearly delineated from the adjacent skin with eschar formation (a collection of dead tissues). Additionally, there was an exudate on the wound’s surface. (**B**) Subgroup IIB showing wound discoloration and necrosis of the eschar. (**C**) Subgroup IIIA showing a red burned area with no scab formation. (**D**) Subgroup IIIB, the burned area was markedly smaller in size and the edges were showing epidermal coverage and hair regrowth.

Naked eye examination of the burned skin revealed that the skin area of the control (group I) showed no apparent change. In group II (subgroup IIA), the burned area was partly covered with a brown dissected scab with exudate formation, and the size of the wound did not exhibit an apparent change (Fig. 9A). After 11 days of burn induction (subgroup IIB), the burned area was covered entirely by scab, and some areas showed necrosis. The size of the wound was nearly the same as in the previous subgroup IIA (Fig. 9B).

In group III (subgroup IIIA), the burned area appeared reddish in colour with less redness around it compared to group II. Additionally, it was dry with neither exudate nor necrosis. There was no scab, and the wound size was apparently smaller than that of the burn model group II (Fig. 9C). Eleven days after the treatment with DSE (subgroup IIIB), the burned area was markedly smaller. The edges showed healing as represented by coverage by the epidermis with hair (Fig. 9D).

**Figure 10:**
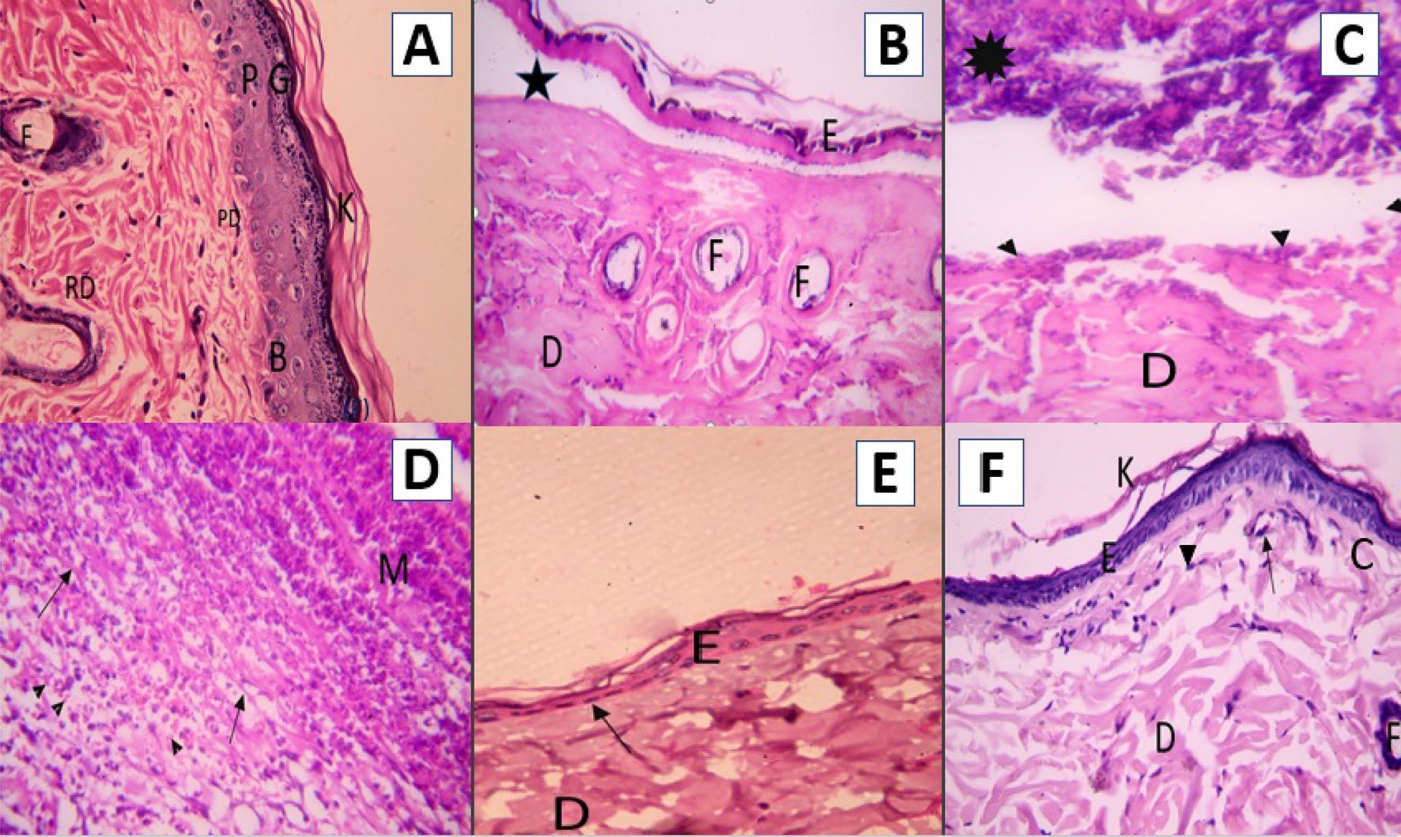
Photomicrographs of a section in the rat’s skin of: (**A**) the control group, demonstrating basal layer (B), prickle cell layers (P), granular layers with basophilic granules (G), and basket weave appearance of the horny layers (K) of the epidermis. Moreover, hair follicles (F) and fine collagen fibers were noticed in the papillary dermis (PD) in addition to thick fibers in the reticular dermis (RD) (400 ×). (**B**) Group IIA illustrating thinning and denudation of epidermis (E) leaving wide space (*) between epidermis and dermis. The dermis contained thick deeply acidophilic and fused denatured collagen fibers (D). A damage of the hair follicles (F) was noticed leaving empty spaces (400 ×). (**C**) Group IIB exposing loss of epidermis (filled triangle) and presence of eschar (*). The dermis contained inflammatory cells and coagulated collagen fibers (400 ×). (**D**) Group IIB displaying deep dermis containing mononuclear inflammatory cells (M), extravasated RBCs (filled triangle), and fine collagen fibers in between (↑) (400 ×). (**E**) Group IIIA illustrating regenerated thin epidermis and flattening of the nuclei of the basal layers (↑). The underlying dermis contained fused hypocellular collagen fibers (D) (400 ×). (**F**) Group IIIB illustrating an apparent increase in the thickness of the epidermis. Overcrowded basal basophilic low columnar cells of stratum basale and keratin layer (K) were revealed. The underlying papillary dermis showed parallel fine collagen fibers (C), flat nuclei of fibroblasts (filled triangle), and blood vessels (↑). The reticular dermis contained thick collagen fibers (D) and hair follicle (F) (400 ×).

Examination with hematoxylin and eosin (H&E) using a brightfield microscope revealed that the skin sections of the control group had two layers of skin: the epidermis and dermis. The epidermis consisted of squamous keratinized epithelium that was stratified, and the dermis consisted of connective tissue. It was divided into two layers without a sharp demarcation: a superficial thin papillary layer and a deep thick reticular layer. Hair follicles were found in the dermis (Fig. 10A).

The H&E examination of thin skin sections of group IIA revealed an exfoliated upper part of the epidermis. The basal layer cells of the epidermis appeared flattened, and the collagen fibers in the upper dermis appeared hypocellular. Moreover, the collagen fibers appeared deeply acidophilic and fused with loss of the distinction between the collagen fibers. Skin appendages such as hair follicles and associated sebaceous glands were damaged, leaving empty spaces (Fig. 10B). The H&E examination of thin skin sections of group IIB showed an epidermal loss, and a scab was formed covering the whole wounded area. The scab showed a deeply acidophilic structure composed of fine fibers and cells. The collagen fibers in the upper dermis appeared hypocellular and deeply acidophilic (Fig. 10C). The deep part of the dermis contained extravasated RBCs, mononuclear inflammatory cellular infiltration, and loose fine collagen fibers (Fig. 10D).

Examination of H&E thin skin sections of group IIIA revealed preservation of a thin epidermis. The epidermis consisted of one layer of flattened keratinocytes with flat nuclei laid on a straight basement membrane. The underlying dermis presented thick and fused collagen fibers (Fig. 10E). H&E staining of group IIIB stained sections revealed epidermal reepithelization, which appeared thick and consisted of stratified squamous epithelium with a thin coating of keratin. The epidermis was relatively thicker than that of the previous subgroup but still thinner than that of the control group, and it showed recesses of the hair follicles. The papillary layer of the dermis was more cellular and had numerous fibroblasts, capillaries, and fine collagen fibers. The reticular dermis revealed large aggregates of collagen bundles that contained thick collagen fibers (Fig. 10F).

**Figure 11:**
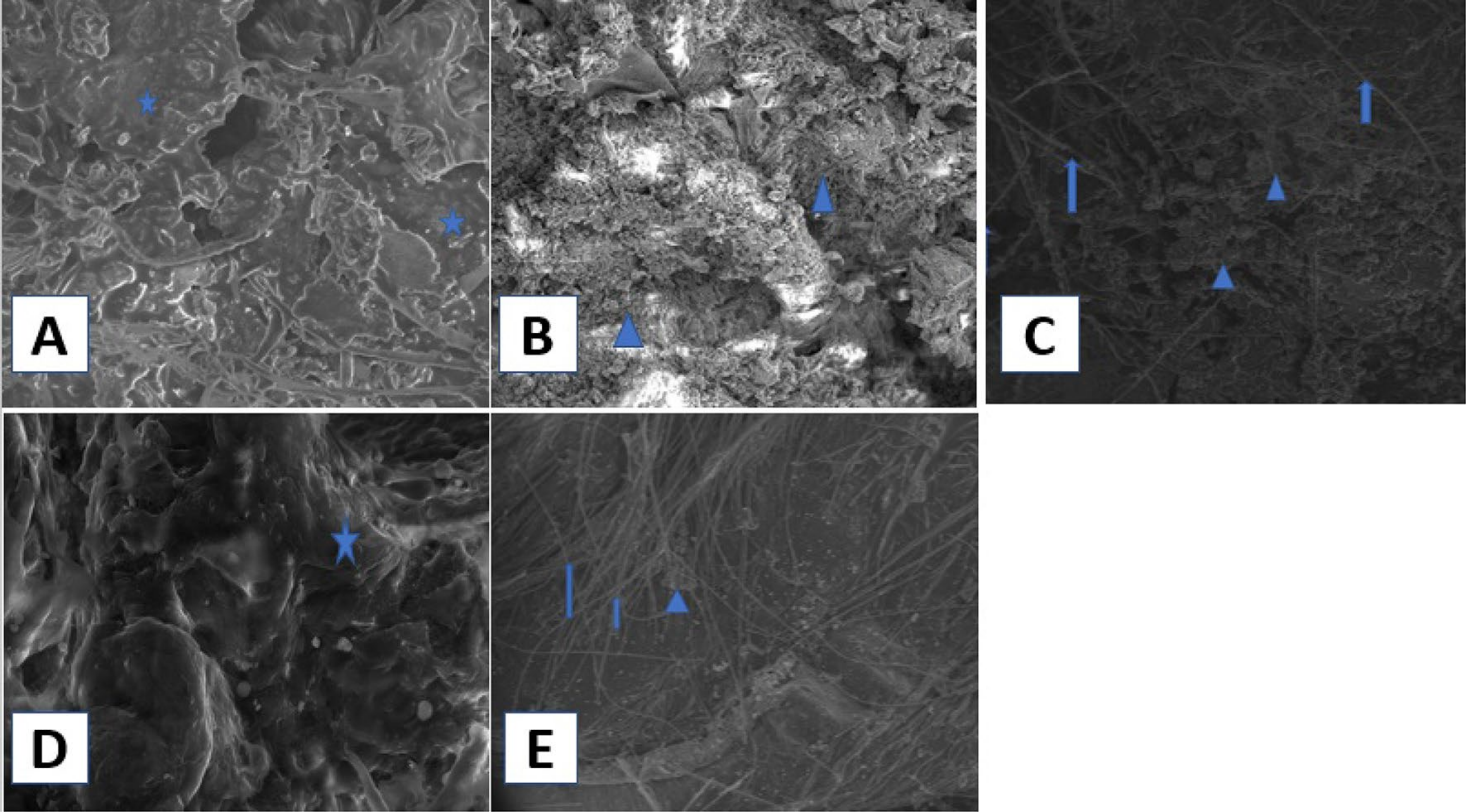
Scanning electron micrograph of the surface layer of the rat’s skin of (**A**) the control group showing that the epidermis (stratum corneum) had layers of overlapped dead skin cells (*) (2500 ×). (**B**) Group IIB showing that the outer layer of the stratum corneum studded with abundant numbers of *P. aeruginosa* bacteria (filled triangle) masking its structure. (2500 ×). (**C**) Group IIB showing collagen fibers (↑) of the dermis with abundant numbers of *P. aeruginosa* bacteria making aggregates like structures (filled triangle) (1000 ×). (**D**) Group IIIB, showing nearly normal stratum corneum (*) comparable to the control group (2500 ×). (**E**) Group IIIB showing the collagen fibers (↑) of the dermis with few numbers of *P. aeruginosa* bacteria making aggregates like structures (filled triangle) in between (1000 ×).

SEM examination of thin skin sections from the control group revealed that the epidermis' outer layer (stratum corneum) had a tough coating made up of overlapping layers of dead skin cells **(**Fig. 11A).

Examination of SEM of thin skin sections of group IIB showed a marked increase in the aggregates like structures formed by *P. aeruginosa* bacteria to a degree higher than that of group IIA. These aggregates, like structures, masked the skin's outer surface and extended deeply into the dermis (Fig. 11B,C).

Examination of SEM of thin skin sections of group IIIB showed a marked reduction of *P. aeruginosa* bacteria on the skin's outer surface, with the appearance of nearly normal stratum corneum to a degree higher than that of group IIIA. The dermis also showed a few aggregates of *P. aeruginosa* bacteria in between collagen fibers (Fig. 11D,E).

### Phytochemical constituents of DSE

**Figure 12:**
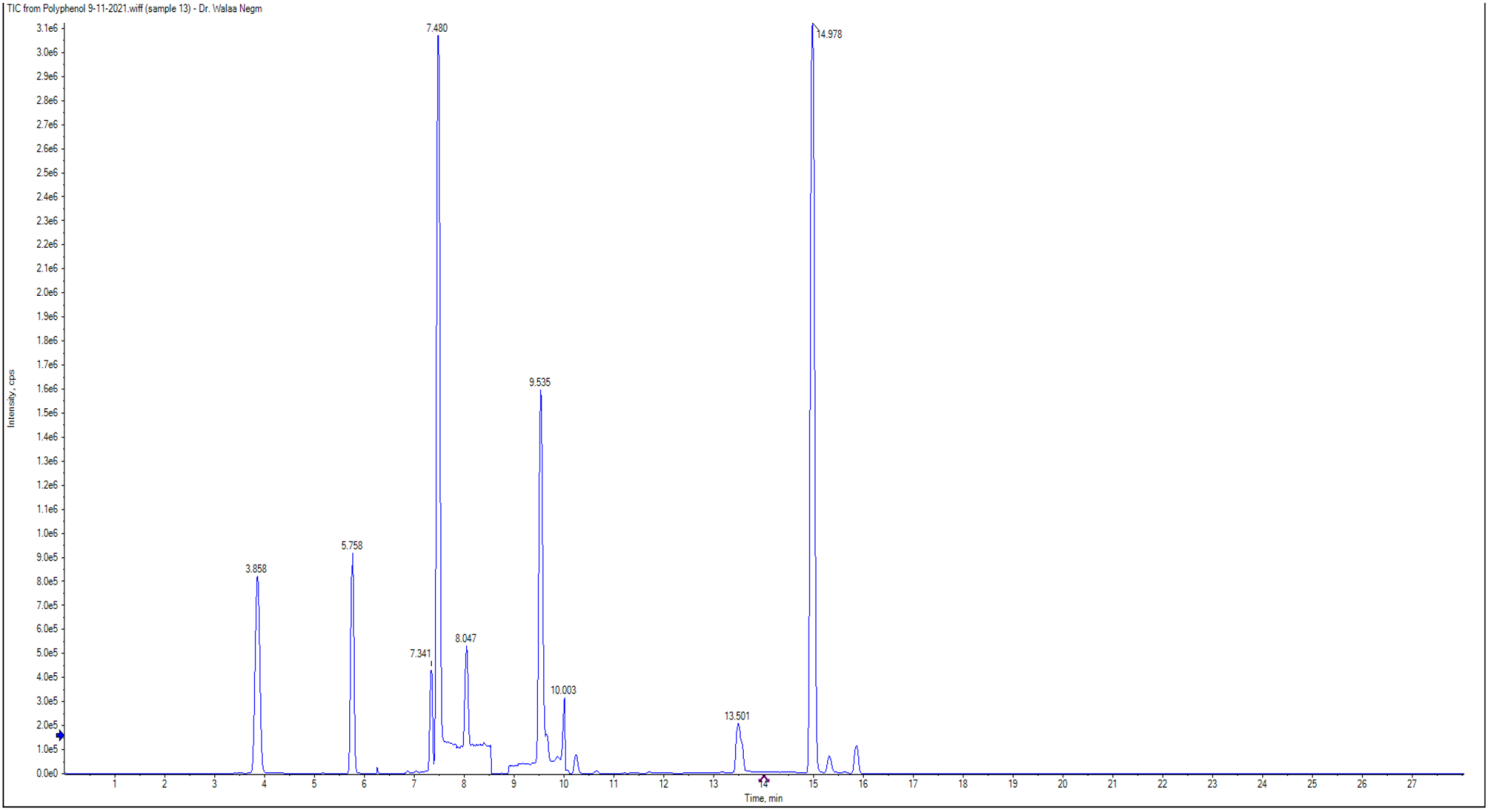
HPLC chromatogram of DSE.

Figure 12 displays the HPLC chromatogram for the identified flavonoids and phenolic compound in DSE. The abundant phenolic compounds in ug/g were ellagic acid (56.20), gallic acid (25.98), vanillin (14.69), methyl gallate (9.98), and chlorogenic acid (9.33), while the identified flavonoid compounds were naringenin (2183.67), apigenin (10.46), kaempferol (0.88), rutin (0.93), and catechin (0.01) (Table 3).

**Table 3:** Chemical composition analysis of phenolic and flavonoid compounds of DSE by HPLC.

Compounds	Samples (conc.ug/g)
**Phenolic**	
Ellagic acid	56.20
Gallic acid	25.98
Vanillin	14.69
Methyl gallate	9.98
Chlorogenic acid	9.33
*O-*coumaric acid	4.70
Ferulic acid	3.20
Cinnamic acid	2.02
Syringic acid	1.35
Caffeic acid	0.67
3,4-Dihydroxybenzoic acid	ND
**Flavonoid**	
Naringenin	2183.67
Apigenin	10.46
Rutin	0.93
Kaempferol	0.88
Catechin	0.01
Querectin	ND
Hesperetin	ND
Myricetin	ND
Daidzein	ND
Luteolin	ND

### Cytotoxicity assay

**Figure 13:**
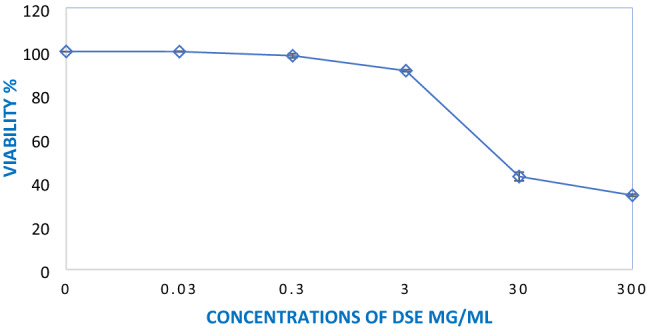
SRB cytotoxicity assay of DSE against HSF normal cell line, after incubation period of 72 h. Different concentrations of 0.03, 0.3, 3, 30 and 300 µg/mL of DSE were used. IC_50s_ values were expressed as mean ± SD of three performed assays.

The sulforhodamine B (SRB) assay was used to determine if DSE was cytotoxic to the human skin fibroblast (HSF) cell line^8,9^. DSE was compared to doxorubicin (a positive control), which had a half-maximal inhibitory concentration (IC_50_) of 4.36 ± 0.52 µg/ml, and DSE had an IC_50_ of 9.4 ± 3.3 µg/ml against the HSF cell line as shown in Fig. 13.

## Discussion

Recently, natural products have been regarded as a vital source for detecting many new therapeutic compounds^10^. *D. spinulosum* exhibited a variety of promising biological activities, including cytotoxic, antioxidant, and antimicrobial potential. This motivated us to undertake this study to search for an alternative effective natural source. Bacteria that are capable of forming biofilms are resistant to many antibiotics. This issue resulted in difficulty in curing biofilm-related infections^11^. It has been established that such bacteria depend mainly on two related phenomena: QS and biofilm formation^12^. The formation of biofilms by pathogenic *P. aeruginosa* isolates is mainly controlled by QS regulatory genes. Thus, anti-quorum sensing substances are usually investigated for inhibition of biofilm formation^13^. It is found that the commercially available anti-quorum sensing compounds could increase the in vitro and in vivo susceptibility of bacteria embedded in biofilms to antibiotics^14^.

In the current study, the anti-quorum sensing and anti-biofilm activities of DSE were investigated against *P. aeruginosa* clinical isolates. The MIC values of DSE were determined, and they ranged from 250 to 1000 µg/ml. The impact of DSE on CSH, EPS quantity, biofilm formation, metabolic activity using MTT, biofilm morphology, and pigment production by *C. violaceum* (ATCC 12472) was assessed at sub-inhibitory concentrations (0.5 MIC values, i.e., at concentrations ranging from 125 to 500 µg/ml) to exclude the effect of DSE on bacterial growth. In addition, its impact on the relative gene expression of the QS and biofilm genes was investigated.

The hydrophobic properties of the bacterial surfaces have a significant role in the bacterial adhesion to various surfaces in addition to the penetration of the host tissues. The hydrophobicity of bacterial surfaces could be measured by calculating HI^15^. We investigated the impact of DSE on CSH of the tested isolates, and a significant reduction (p < 0.05) in the values of HI was noticed in 45.7% of the tested isolates after treatment with DSE.

Polysaccharides present in the biofilm matrix could provide various benefits to the bacterial cells embedded in biofilms, like adhesion and protection. They can act like glue, which permits the bacterial cells to adhere to each other as well as different surfaces. Moreover, polysaccharides could protect the cells in biofilms from desiccation and immune effectors^16^. Herein, we found that DSE exhibited a significant reduction (p < 0.05) in the extracellular polysaccharides' quantity in 51.4% of *P. aeruginosa* isolates. The primary mechanism for such a reduction in EPS production after treatment with DSE needs further investigation.

There are two critical steps in biofilm production. The first step involves bacterial adhesion to different surfaces, which provides the groundwork for further biofilm development.

The second step involves the interactions between cells. At this stage, bacteria form microcolonies to construct a multilayer structure for biofilm development^17^. In the current study, the biofilm inhibition by DSE was evaluated using the crystal violet test. DSE decreased the percentage of *P. aeruginosa* isolates strongly and moderately producing biofilms from 77.1 to 34.3%. Determination of CFU by agar plating is considered as the gold standard method for quantification of biofilm^18^. The main principle of this test is to quantify the living bacterial cells using an agar plate as they grow forming colonies that could be counted^19^. There was a significant reduction in the number of CFU/ml in 34.3 % of the isolates after treatment with DSE.

MTT assay was used to detect the effect of DSE on the viability of bacteria embedded in the biofilm. Viable bacterial cells with active metabolism will have the capability to convert MTT into formazan (purple colour) with an absorbance of 550 nm. On the other hand, dead bacterial cells lose this capability. So, purple colour formation is a marker for viable cells only^20^. A non-significant change (p > 0.05) in the bacterial cells' viability was observed after treatment with DSE. To better understand the effect of DSE on biofilm formation by *P. aeruginosa* isolates, biofilm morphology was inspected using a compound bright field microscope and SEM. Microscopical examination confirmed DSE's biofilm inhibitory action against the tested isolates, as a significant reduction in the biofilms formed by *P. aeruginosa* isolates was observed after treatment with DSE.

Violacein is a dark purple indole derivative agent. Its production is essential for QS and hence biofilm formation. A reference strain, *C. violaceum* (ATCC 12472), is known for its ability to produce violacein, and thus, it is widely utilized for QS studies^21^. DES showed an anti-quorum sensing activity against *C. violaceum* (ATCC 12472) as it inhibited violacein production in a concentration-dependent manner.

qRT-PCR was utilized to inspect the impact of DSE on QS and biofilm formation genes in 15 *P. aeruginosa* isolates. Our results revealed that treatment with DSE decreased the expression of *las*I, *las*R, *rhl*I, *rhl*R, and *ndv*B genes in 46.67%, 40%, 60%, 53.33%, and 66.67% of the selected *P. aeruginosa* isolates, respectively.

The current study produced a partial thickness skin burn injury in rat groups II and III by scalding with hot water (99 °C) for 3 s^22^. Moreover, a mould was created to restrict the burn injury to a small uniform area of 1.1% of the total body surface area in the back to avoid shock and the death of rats^23^. Examination of group II (burn model) sections stained by H&E showed a range of epidermal thinning, exfoliation, and necrosis. This came per Tanaka et al.^24^. In addition, Yang et al.^25^ found that the skin epidermis in deep second-degree burns was almost entirely dissected from the underlying dermis.

Meanwhile, the underlying dermis in the present study showed collagen denaturation in the form of swollen, fused, and closely packed collagen fibers. Similarly, Park et al.^26^ noticed that when the skin was thermally damaged, the fibers had a glass-like appearance from the dense coagulation of collagen. On the other hand, Tanaka et al.^24^ detected a loss of boundaries between collagen fibers and attributed this to water leakage from capillaries, causing edema and fusion of collagen fibers. The hair follicles with their associated glands were completely damaged in group II. Younan et al.^27^ detected the same finding.

SEM examination of group II showed biofilm formation. Chu et al.^26^ stated that owing to the ability of *P. aeruginosa* to create biofilms on both biotic and abiotic surfaces within hours, and it could form biofilms within the burn eschar (a collection of dead tissues). These biofilms could serve as a nidus for long-term wound infection and transplant failure. In addition, *P. aeruginosa* also expresses various proteases, including collagenase, which may aid it in entering burned skin^22^. Furthermore, the biofilm infection causes an inflammatory response in the local burn skin infected with *P. aeruginosa*, as evidenced by increased circulating neutrophils and increased myeloperoxidase activity. The inflammatory cells seen in H&E -stained tissue sections mainly were neutrophils.

Meanwhile, in group III, treatment with DSE resulted in epidermal regeneration, which was started by the migration of cells from the basal layer of adjacent healthy skin. DSE significantly improved wound contracture and healing. Furthermore, it appeared to improve the healing process by regenerating the epidermis and reducing inflammatory cell infiltration associated with collagen fiber deposition compared to the infected group. It also showed nearly intact hair follicles. This comes in agreement with Negm et al.^7^, who stated that DSE showed higher activity against Gram-negative bacteria, like *E. coli* and *P. aeruginosa*, than Gram-positive bacteria.

## Materials and methods

### Bacteria

Thirty-five *P. aeruginosa* isolates were collected from Tanta University hospitals. These clinical isolates were subjected to microscopical examination, and they were identified using standard biochemical tests according to MacFaddin^28^. *Pseudomonas aeruginosa* ATCC 27853 was utilized as the standard isolate.

### Plant material

*Dioon spinulosum* Dyer Ex Eichler leaves were selected from El Abd Garden in Giza city on January 14th, 2017. The permission for plant collection was obtained from the landowner, Mr. Rabea Sharawy. Then, Dr. Esraa Ammar (a plant ecology lecturer at Botany Department, Faculty of Science, Tanta University) and Mr. Rabea Sharawy (an agronomist and palm researcher were) kindly identified the plant. A voucher specimen number: PGG-002 was deposited at Pharmacognosy Department herbarium, Faculty of Pharmacy, Tanta University.

One kilogram of shade-dried powdered leaves of *D. spinulosum* was extracted by cold maceration with MeOH (3L×3, 48 h each) then concentrated under vacuum using a rotary evaporator at 45 °C to yield the crude extract (87.2 g) that were used for biological investigations.

### Chemicals

All the utilized chemicals were of analytical grade, obtained from Merck (New Jersey, United States). The standard phenolic and flavonoid compounds used were gallic acid, methyl gallate, chlorogenic acid, vanillin, caffeic acid, syringic acid, ferulic acid, ellagic acid, 3,4-dihydroxybenzoic acid, *O*-coumaric acid, cinnamic acid, apigenin, rutin, myricetin, catechin, quercetin, naringenin, hesperetin, daidzein, luteolin, and kaempferol.

### Determination of MICs of DSE against the tested isolates

The MICs of DSE were determined by the broth microdilution method^29^. Each plate contained a positive control (untreated bacteria) and negative control (non-inoculated well).

All the following experiments were performed before and after treatment of the tested isolates with sub-inhibitory concentrations of DSE (0.5 MIC values).

### Determination of CSH

It was determined in the tested isolates before and after treatment with DSE (with concentrations of 0.5 MIC values that ranged from 125 to 500 µg/ml) according to the method previously explained^30^. After centrifugation of the bacterial suspensions, the pellets were resuspended in phosphate urea magnesium sulfate buffer (PUM buffer, pH 6.9). Then, several volumes of *n*-hexane (0.3, 0.9, 1.2, and 1.8 ml) were added to 4.8 ml of each bacterial suspension in the PUM buffer and vortexed. The suspension was allowed to stand for complete phase separation. Finally, the absorbance of the bacterial isolates that remained in the aqueous phase was measured at 540 nm. The HI was determined as follow:

$$HI = (A540\,\, control - A540\,\, test)/A540\,\, control$$

### Quantification of EPS

EPS extraction and quantification were carried out as previously described^31^. Briefly, the tested isolates were overnight grown at 37°C in Luria-Bertani (LB) broth with (treated isolates) and without (untreated isolates) DSE (using concentrations ranging from 125 to 500 µg/ml). After incubation, they were centrifuged, and the pellets were resuspended in phosphate-buffered saline (PBS) buffer and centrifuged again. Then, ethyl alcohol was added to the supernatant with an equal volume and centrifuged. Finally, 1 ml of the EPS solution was thoroughly mixed with an equal volume of cold 5% phenol plus 5 ml concentrated sulfuric acid. The optical intensity (OD) of the produced red colour was measured at 490 nm, and the percentages of inhibition of EPS were calculated.

### In vitro biofilm inhibition assay

Biofilm formation was examined in the tested isolates before and after treatment with DSE (with concentrations ranging from 125 to 500 µg/ml) using the crystal violet test^32,33^. The OD at 490 nm was measured using an ELISA reader (Sunrise Tecan, Grodig, Austria). The tested isolates were characterized into four groups based on their ODs as follows:

i. NBP: non-biofilm producer (ODc < OD < 2 ODc)
ii. WBP: weak biofilm producer (2 ODc < OD < 4 ODc)
iii. MBP: moderate biofilm producer (4 ODc < OD < 6 ODc)
iv. SBP: strong biofilm producer (6 ODc < OD)

The cut-off OD (ODc) is the mean OD plus 3 SD of the negative control.

### Determination of CFU

The formed biofilms in the wells of the microtitration plates were washed twice with PBS so that the loosely attached cells were removed. Then, the biofilms were scraped off the wells through vigorous scraping using a pipette tip after adding 200 μl PBS. They were then vortexed in order to homogenize the biofilm. After that, they were serially diluted, and 100 μl from each dilution was plated on Muller-Hinton agar (MHA) plates. After overnight incubation, the CFU were counted^18^.

### Estimation of the metabolic activity of biofilm by MTT assay

The MTT colorimetric assay was performed as previously described^30,34^. Briefly, the bacterial suspensions were overnight incubated with (treated isolates) and without (untreated isolates) DSE (using concentrations ranging from 125 to 500 µg/ml). To each well of the microtitration plate, 150 µl of PBS and 50 µl of MTT solution were added. Then, the MTT solution was withdrawn after 2 h, and 150 µl of dimethyl sulphoxide (DMSO) was added instantly as a solubilizing agent. Finally, the absorbance was measured at a wavelength of 550 nm by an ELISA reader (Sunrise Tecan, Grodig, Austria).

### Examination of biofilm morphology

The effect of DSE was tested against biofilm formation by *P. aeruginosa* isolates on glass surfaces as previously described^35^. Biofilm formed by *P. aeruginosa* with (treated isolates) and without (untreated isolates) DSE (using concentrations ranging from 125 to 500 µg/ml) was visualized by a compound bright field microscope (Labomed, California, America) using 40× magnification after staining with crystal violet.

### Scanning electron microscope (SEM)

SEM was used to examine the bacterial biofilms formed on the surfaces of cover glass with (treated isolates) and without (untreated isolates) DSE (using concentrations ranging from 125 to 500 µg/ml) as described previously^36,37^, and examined using an S‐3400N scanning electron microscope (Hitachi, Tokyo, Japan).

### Quantification of inhibition of violacein production

It was performed as described by Ganesh and Rai^31^. DSE (with concentrations ranging from 62.5 to 1000 µg/ml) was added to a 10 µl suspension of *C. violaceum* (ATCC 12472) in LB broth and incubated overnight at 30 °C. Centrifugation was performed after incubation, and 500 µl of DMSO was added to each pellet and vortexed vigorously till the extraction of violacein (purple colour). The extracted violacein (200 µl) was transferred into a 96 well microtitration plate and quantified using an ELISA reader (Sunrise Tecan, Grodig, Austria) at 585 nm. The control was the bacterial culture of *C. violaceum* in LB broth without the DSE.

### qRT-PCR

The expression of 4 quorum sensing genes (*las*I*, las*R*, rhl*I*, rhl*R)^38^, and the biofilm gene (ndvB) were identified using qRT-PCR, and the *16S rRNA* gene was utilized as the housekeeping gene^39^. All the experiments were conducted in triplicate, and the results were expressed as mean± SD values. According to the manufacturer's instructions, after extraction of the total RNA, using the GeneJET RNA purification kit, K0731 (Thermo Scientific, Waltham, USA), it was retrotranscribed into cDNA by using a power cDNA synthesis kit (iNtRON Biotechnology, Seongnam, Korea). The utilized primers are shown in Table S1. The amplification was performed using the Power SYBR^®^ Green master mix (Thermo SCIENTIFIC, Waltham, USA) and the Rotor-Gene Q 5plex apparatus (Qiagen, Hilden, Germany). The relative gene expression was calculated by using the 2^−ΔΔCt^ method^40^. Non-treated isolates were used as control samples (i.e., expression set to 1).

### In vivo biofilm inhibition assay

All of the animals were purchased and grown at Ain Shams University's Medical Research Center. Twenty-five adult female albino rats of an average weight of 200–250 g were utilized. The animal experiment was carried out at the Ain Shams University Faculty of Medicine's Research Center Institute (MASRI). It was approved by the Faculty of Medicine, Ain Shams University Research Ethics Committee (FMASU REC) organized and run under the International Council on Harmonization (ICH) and Islamic Organization for Medical Science (IOMS) guidelines, as well as the US Office for Human Research Protections and US Code of Federal Regulations and is covered by Federal Wide Assurance N.FWA 00017585.

### The Burn model

Intraperitoneal injection of 40 mg/kg pentobarbital sodium was used to anesthetize the rats^41^. The dorsal hair of the rats was removed from an area of 4 cm^2^ in the middle of their backs, 3 cm away from the neck, first with clippers and then through the application of one^®^ depilatory cream. A mould was applied to the shaved area of the back of the rats. Hot water at 99 °C was poured into the mould for 3 s to induce a partial-thickness burn^22^. The rats were divided into three groups:

Group I: consisted of 5 rats, which served as the control group.

Group II (inoculated untreated burn group): consisted of 10 rats. The same procedure was applied as previously described in the burn model; then, the burning surface was inoculated with 30 µl of a suspension of 1 ×10^8^ CFU/ml of *P. aeruginosa* in the center of each wound. Then, a pipette tip was used to spread it to cover the entire burn area^42^. This group is further divided into:

Group IIA: consisted of 5 rats sacrificed 3 days after creating the inoculated burn.

Group IIB: consisted of 5 rats, sacrificed 11 days after creating the inoculated burn.

Group III (inoculated treated burn group): consisted of 10 rats. The same procedures were applied as in group II, then a solution of DSE was applied daily to the burn area. This group is further divided into:

Group IIIA: consisted of 5 rats, sacrificed 3 days after creating the inoculated burn.

Group IIIB: consisted of 5 rats, sacrificed 11 days after creating the inoculated burn.

### Histological studies

Five micrometer paraffin sections were prepared and stained with H&E and examined by a brightfield microscope to show the histological details^43^. Moreover, the burn wound surface-associated *P. aeruginosa* biofilms were examined using SEM^43^.

### Analyses of phenolics and flavonoids by HPLC

An Agilent 1260 Infinity HPLC Series (Agilent, Santa Clara, CA, USA), equipped with a Quaternary pump and a Zorbax Eclipse Plus C18 column (100 mm × 4.6 mm i.d.) (Agilent Technologies, Santa Clara, CA, USA), was operated at 30 °C. The separation was achieved using a ternary linear elution gradient with (A) HPLC grade water, 0.2% H_3_PO_4_ (v/v), (B) methanol, and (C) acetonitrile. The injectable volume was 20 μL. A VWD detector was set at 284 nm to identify the phenolic compounds in the DSE^44,45^.

### Cytotoxicity assay

The SRB test was used to measure HSF cell viability^8,9^. Aliquots of 100 µl cell suspension (5×10^3^ cells) were incubated in full medium for 24 h in 96-well plates. A different aliquot of 100 µl medium and the DSE at varied doses was utilized to treat the cells. The cells were fixed by replacing the medium with 150 µl of 10% trichloroacetic acid (TCA) and incubated for 60 min at 4 °C after 72 h of exposure to DSE. The HSF cells were rinsed five times with distilled water, and liquors of 70 μl SRB solution (0.4% w/v) were added and kept in a dark place for 10 min at room temperature. Plates were washed three times with 1% acetic acid and left to dry overnight in the air. Finally, 150 µl of Tris base solution (10 mM) was added to dissolve the protein-bound SRB stain, and the absorbance was measured at 540 nm using an ELISA reader.

### Statistical analysis

All the performed assays were carried out in triplicates, and the results were presented as mean ± SD. The statistical significance of the results was determined by one-way ANOVA using SPSS software (IBM, USA), and only results with a p < 0.05 were considered significant. Statistical analysis:

### Ethical approval

All experiments were performed in accordance with the relevant guidelines and regulations. All experiments and protocols were approved by Ain Shams University and Tanta university. The animal experiment was carried out at the Ain Shams University Faculty of Medicine's Research Center Institute (MASRI). The animal experiments were approved by the FMASU REC organized and run under the International Council on Harmonization (ICH) and Islamic Organization for Medical Science (IOMS) guidelines, as well as the US Office for Human Research Protections and US Code of Federal Regulations and is covered by Federal Wide Assurance N.FWA 00017585. All reported methods are in accordance with ARRIVE guidelines.

## Conclusion

The current study revealed the potential in vitro and in vivo anti-biofilm and anti-quorum sensing activities of DSE as it exhibited efficient activity against *P. aeruginosa* clinical isolates. To our knowledge, this is the first time to report the ability of DSE to reduce biofilm formation by *P. aeruginosa* via downregulation of quorum sensing genes. This finding could help in the reduction of our significant dependence on antibiotics. Moreover, this could help us efficiently handle the biofilm-related infections caused by such opportunistic pathogens. Thus, further studies are necessary to examine the possible inhibitory effects of DSE on biofilms formed by other pathogens. In addition, preclinical and clinical studies are required to enable its practical implementation in managing *P. aeruginosa* biofilm-related infections.

## Supplementary Information

Supplementary Information.

## Data Availability

The data supporting this study are available upon request.

## References

[1] El-Sayed NR, Samir R, Abdel-Hafez JM, Ramadan MA (2020). Olive leaf extract modulates quorum sensing genes and biofilm formation in multi-drug resistant Pseudomonas aeruginosa. Antibiotics.

[2] Su S, Hassett DJ (2012). Anaerobic Pseudomonas aeruginosa and other obligately anaerobic bacterial biofilms growing in the thick airway mucus of chronically infected cystic fibrosis patients: An emerging paradigm or “Old Hat”?. Expert Opin. Ther. Targets.

[3] Sugano M (2016). Potential effect of cationic liposomes on interactions with oral bacterial cells and biofilms. J. Liposome Res..

[4] Wagner S (2016). Novel strategies for the treatment of Pseudomonas aeruginosa infections. J. Med. Chem..

[5] Lee J, Zhang L (2015). The hierarchy quorum sensing network in Pseudomonas aeruginosa. Protein Cell.

[6] Negm W, Abo El-Seoud K, Kabbash A, El-Aasr M (2020). Investigation of the biological activity some gymnosperm plants belong to cycadales order. J. Adv. Med. Pharmaceut. Res..

[7] Negm WA, Abo El-Seoud KA, Kabbash A, Kassab AA, El-Aasr M (2020). Hepatoprotective, cytotoxic, antimicrobial and antioxidant activities of Dioon spinulosum leaves Dyer Ex Eichler and its isolated secondary metabolites. Nat. Prod. Res..

[8] Skehan P (1990). New colorimetric cytotoxicity assay for anticancer-drug screening. JNCI.

[9] Allam RM (2018). Fingolimod interrupts the cross talk between estrogen metabolism and sphingolipid metabolism within prostate cancer cells. Toxicol. Lett..

[10] Atanasov AG, Zotchev SB, Dirsch VM, Supuran CT (2021). Natural products in drug discovery: Advances and opportunities. Nat. Rev. Drug Discovery.

[11] Sharma D, Misba L, Khan AU (2019). Antibiotics versus biofilm: An emerging battleground in microbial communities. Antimicrob. Resist. Infect. Control.

[12] Zhao X, Yu Z, Ding T (2020). Quorum-sensing regulation of antimicrobial resistance in bacteria. Microorganisms.

[13] Singh VK, Mishra A, Jha B (2017). Anti-quorum sensing and anti-biofilm activity of Delftia tsuruhatensis extract by attenuating the quorum sensing-controlled virulence factor production in Pseudomonas aeruginosa. Front. Cell. Infect. Microbiol..

[14] Jiang Y, Geng M, Bai L (2020). Targeting biofilms therapy: Current research strategies and development hurdles. Microorganisms.

[15] Krasowska A, Sigler K (2014). How microorganisms use hydrophobicity and what does this mean for human needs?. Front. Cell. Infect. Microbiol..

[16] Limoli DH, Jones CJ, Wozniak DJ (2015). Bacterial extracellular polysaccharides in biofilm formation and function. Microbio. Spectrum.

[17] Olivares E (2020). Clinical impact of antibiotics for the treatment of Pseudomonas aeruginosa biofilm infections. Front. Microbiol..

[18] Thieme, L. *et al.* Adaptation of the start-growth-time method for high-throughput biofilm quantification. *Front. Microbiol.* 2395 (2021). 10.3389/fmicb.2021.631248 PMC8428173 34512560

[19] Wilson, C. *et al.* Quantitative and qualitative assessment methods for biofilm growth: A mini-review. *Res. Rev. J. Eng. Technol.* **6** (2017). PMC6133255 30214915

[20] Benov L (2019). Effect of growth media on the MTT colorimetric assay in bacteria. PLoS ONE.

[21] Kanelli M (2018). Microbial production of violacein and process optimization for dyeing polyamide fabrics with acquired antimicrobial properties. Front. Microbiol..

[22] Brandenburg KS (2019). Development of Pseudomonas aeruginosa biofilms in partial-thickness burn wounds using a Sprague-Dawley rat model. J. Burn Care Res..

[23] Tavares Pereira, D. d. S., Lima-Ribeiro, M. H. M., de Pontes-Filho, N. T., Carneiro-Leão, A. M. d. A. & Correia, M. T. d. S. Development of animal model for studying deep second-degree thermal burns. *J. Biomed. Biotechnol.* **2012** (2012). 10.1155/2012/460841 PMC3379528 22736951

[24] Tanaka R (2013). In vivo visualization of dermal collagen fiber in skin burn by collagen-sensitive second-harmonic-generation microscopy. J. Biomed. Opt..

[25] Yang Y, Zhang W, Li Y, Fang G, Zhang K (2014). Scalded skin of rat treated by using fibrin glue combined with allogeneic bone marrow mesenchymal stem cells. Ann. Dermatol..

[26] Park BH, Saxer CE, Srinivas SM, Nelson JS, de Boer JF (2001). In vivo burn depth determination by high-speed fiber-based polarization sensitive optical coherence tomography. J. Biomed. Opt..

[27] Younan G (2010). The inflammatory response after an epidermal burn depends on the activities of mouse mast cell proteases 4 and 5. J. Immunol..

[28] MacFaddin, J. Biochemical Tests for Identification of Medical Bacteria, Williams and Wilkins. *Philadelphia, PA* **113** (2000).

[29] Wayne, A. Clinical and Laboratory Standards Institute; CLSI. 2017. Performance standards for antimicrobial susceptibility testing. 20th Informational Supplement. *CLSI document* (2017).

[30] El-Banna T, Abd El-Aziz A, Sonbol F, El-Ekhnawy E (2019). Adaptation of Pseudomonas aeruginosa clinical isolates to benzalkonium chloride retards its growth and enhances biofilm production. Mol. Biol. Rep..

[31] Ganesh PS, Rai VR (2018). Attenuation of quorum-sensing-dependent virulence factors and biofilm formation by medicinal plants against antibiotic resistant Pseudomonas aeruginosa. J. Tradit. Complement. Med..

[32] Elekhnawy EA, Sonbol FI, Elbanna TE, Abdelaziz AA (2021). Evaluation of the impact of adaptation of Klebsiella pneumoniae clinical isolates to benzalkonium chloride on biofilm formation. Egypt. J. Med. Hum. Genet..

[33] Yang H, Zhang H, Wang J, Yu J, Wei H (2017). A novel chimeric lysin with robust antibacterial activity against planktonic and biofilm methicillin-resistant Staphylococcus aureus. Sci. Rep..

[34] Trafny EA, Lewandowski R, Zawistowska-Marciniak I, Stępińska M (2013). Use of MTT assay for determination of the biofilm formation capacity of microorganisms in metalworking fluids. World J. Microbiol. Biotechnol..

[35] Attallah NG (2021). Antibacterial activity of Boswellia sacra Flueck. Oleoresin extract against Porphyromonas gingivalis periodontal pathogen. Antibiotics.

[36] Attallah NGM (2021). Elucidation of phytochemical content of Cupressus macrocarpa leaves: in vitro and in vivo antibacterial effect against methicillin-resistant Staphylococcus aureus clinical isolates. Antibiotics.

[37] Elekhnawy E, Sonbol F, Abdelaziz A, Elbanna T (2021). An investigation of the impact of triclosan adaptation on Proteus mirabilis clinical isolates from an Egyptian university hospital. Braz. J. Microbiol..

[38] Rajkumari J (2019). Anti-quorum sensing and anti-biofilm activity of 5-hydroxymethylfurfural against Pseudomonas aeruginosa PAO1: Insights from in vitro, in vivo and in silico studies. Microbiol. Res..

[39] Li P-J (2015). Effect of prolonged radiotherapy treatment time on survival outcomes after intensity-modulated radiation therapy in nasopharyngeal carcinoma. PLoS ONE.

[40] Livak KJ, Schmittgen TD (2001). Analysis of relative gene expression data using real-time quantitative PCR and the 2− ΔΔCT method. Methods.

[41] Gaertner, D., Hallman, T., Hankenson, F. & Batchelder, M. Anesthesia and analgesia for laboratory rodents, p 239–297. *Anesthesia and analgesia in laboratory animals. London (UK): Elsevier* (2008).

[42] Andersson MÅ, Madsen LB, Schmidtchen A, Puthia M (2021). Development of an experimental ex vivo wound model to evaluate antimicrobial efficacy of topical formulations. Int. J. Mol. Sci..

[43] Brandenburg KS (2019). Formation of Pseudomonas aeruginosa biofilms in full-thickness scald burn wounds in rats. Sci. Rep..

[44] Behiry SI (2019). Antifungal and antibacterial activities of *Musa paradisiaca* L. peel extract: HPLC analysis of phenolic and flavonoid contents. Processes.

[45] Ashmawy NA (2020). Eco-friendly wood-biofungicidal and antibacterial activities of various *Coccoloba uvifera* L. leaf extracts: HPLC analysis of phenolic and flavonoid compounds. BioResources.

