# Supplementary material for: Histological assessment, anti-quorum sensing, and anti-biofilm activities of Dioon spinulosum extract: in vitro and in vivo approach
Source: Sci Rep. 2022 Jan 7;12:180. doi: 10.1038/s41598-021-03953-x (PMC8742103; doi:10.1038/s41598-021-03953-x)
Supplement: Supplementary file 1 — Supplementary Information. [file 41598_2021_3953_MOESM1_ESM.docx]

Histological Assessment, Anti-quorum Sensing, and Anti-biofilm Activities of *Dioon spinulosum* Extract: *in vitro* and *in vivo* Approach.

**Table S1.** Sequences of the primers used in RT–PCR

| **Tested genes** | **Primer direction** | **Sequence** |
| --- | --- | --- |
| *las*I | Forward | 5'-GTGACGGTAACCACCGTAGG-3ˋ |
|  | Reverse | 5'-CTGGGTCTTGGCATTGAGTT-3ˋ |
| *las*R | Forward | 5'-CTGTGGATGCTCAAGGACTAC-3ˋ |
|  | Reverse | 5'-AACTGGTCTTGCCGATGG-3ˋ |
| *rhl*I | Forward | 5'-AAGGACGTCTTCGCCTACCT-3ˋ |
|  | Reverse | 5'-GCAGGCTGGACCAGAATATC-3ˋ |
| *rhl*R | Forward | 5'-CATCCGATGCTGATGTCCAACC-3ˋ |
|  | Reverse | 5-ATGATGGCGATTTCCCCGGAAC-3′ |
| *ndv*B | Forward | 5'**-**GGCCTGAACATCTTCTTCAC-3′ |
|  | Reverse | 5'**-**GATCTTGCCGACCTTGAAGA-3′ |
| *16S rRNA* | Forward | 5'-TTGGGAGGAAGGGCATTAAC-3ˋ |
|  | Reverse | 5'-CGCTTGCACCCTCTGTATTA-3ˋ |
